# Supplementary material for: Tumor suppressor Tsc1 is a new Hsp90 co‐chaperone that facilitates folding of kinase and non‐kinase clients
Source: EMBO J. 2017 Nov 10;36(24):3650–65. doi: 10.15252/embj.201796700 (PMC5730846; doi:10.15252/embj.201796700)
Supplement: Supplementary file 1 — Appendix [file EMBJ-36-3650-s001.pdf]

Appendix Table of Content

Appendix Table S1 page 2-8
Appendix References page 9

Table S1. List of antibodies, commercial assays, mammalian cell lines, yeast strains, oligonucleotides and recombinant DNA plasmids. Primer sequences with restriction sites in red, epitope sequences highlighted in green and mutation sites in blue.

REAGENT or RESOURCE	SOURCE	IDENTIFIER
Antibodies		
Anti-rabbit Aha1 (1:4000)	StressMarq Biosciences	Cat# SPC-183 RRID:AB_1944037
Anti-rabbit Hsp70 (1:20,000)	StressMarq Biosciences	Cat# SPC-103 RRID:AB_2570584
Anti-rabbit Cdc37 (1:4000)	StressMarq Biosciences	Cat# SPC-142 RRID:AB_2570605
Anti-rabbit FLAG tag (1:8000)	Thermo Scientific	Cat# PA1-984B RRID:AB_347227
Anti-mouse 6x-His (HIS.H8) (1:10,0000)	Thermo Scientific	Cat# MA1-21315 RRID:AB_557403
Anti-mouse CFTR (M3A7) (1:1000)	EMD Millipore	Cat# 05-583 RRID:AB_309819
Anti-rat Hsp90 (16F1) (1:8000)	Enzo Life Sciences	Cat# ADI-SPA-835 RRID:AB_11181205
Anti-rabbit p23 (1:2000)	Enzo Life Sciences	Cat# ADI-SPA-670 RRID:AB_10617386
Anti-mouse GAPDH (1D4) (1:10,000)	Enzo Life Sciences	Cat# ADI-CSA-335 RRID:AB_10617247

Anti-rabbit FLCN (D14G9)<br><br>(1:4000)	Cell Signaling Technology	Cat# 3697 RRID:AB_2231646
Anti-rabbit HOP (D10E2)<br><br>(1:2000)	Cell Signaling Technology	Cat# 5670 RRID:AB_10828369
Anti-rabbit PP5<br><br>(1:1000)	Cell Signaling Technology	Cat# 2289 RRID:AB_2168757
Anti-rabbit Phospho-Akt S473 (D9E)<br><br>(1:2000)	Cell Signaling Technology	Cat# 2289 RRID:AB_2315049
Anti-mouse Akt (2H10)<br><br>(1:4000)	Cell Signaling Technology	Cat# 2967 RRID:AB_331160
Anti-rabbit GR (D6H2L)<br><br>(1:2000)	Cell Signaling Technology	Cat# 12041 RRID:AB_2631286
Anti-rabbit Phospho-GR S211<br><br>(1:2000)	Cell Signaling Technology	Cat# 4161 RRID:AB_2155797
Anti-rabbit HA-Tag (C29F4)<br><br>(1:2000)	Cell Signaling Technology	Cat# 3724 RRID:AB_1549585
Anti-rabbit ULK1 (D8H5)<br><br>(1:1000)	Cell Signaling Technology	Cat# 8054 RRID:AB_11178668
Anti-rabbit ERα (D6R2W)<br><br>(1:2000)	Cell Signaling Technology	Cat# 13258 RRID: AB_2632959
Anti-rabbit TSC1<br><br>(1:1000)	Cell Signaling Technology	Cat# 4906 RRID:AB_2209790

Anti-rabbit TSC2 (D57A9)<br><br>(1:1000)	Cell Signaling Technology	Cat# 3990 RRID:AB_2209986
Anti-rabbit Src (32G6)<br><br>(1:2000)	Cell Signaling Technology	Cat# 2123 RRID:AB_2106047
Anti-rabbit Phospho-Src Y416<br><br>(1:2000)	Cell Signaling Technology	Cat# 2101 RRID:AB_331697
Anti-rabbit c-Abl<br><br>(1:1000)	Cell Signaling Technology	Cat# 2862 RRID:AB_2257757
Anti-rabbit c-Abl Y89 (61A6)<br><br>(1:1000)	Cell Signaling Technology	Cat# 3098 RRID:AB_2220865
Anti-mouse Ubiquitin (P4D1)<br><br>(1:1000)	Santa Cruz Biotech	Cat# sc-8017 RRID:AB_628423
Anti-rabbit Raf-1 (C-12)<br><br>(1:4000)	Santa Cruz Biotech	Cat# sc-133 RRID:AB_632305
Anti-rabbit Cdk4 (H-22)<br><br>(1:4000)	Santa Cruz Biotech	Cat# sc-601 RRID:AB_631221
Anti-rabbit ErbB2/Neu (C-18)<br><br>(1:1000)	Santa Cruz Biotech	Cat# sc-284 RRID:AB_632013
Anti-rabbit Sba1<br><br>(1:8000)	The Institute of Cancer Research, London	
Anti-mouse secondary<br><br>(1:4000)	Santa Cruz Biotech	Cat# sc-2005 RRID:AB_631736

Anti-rabbit secondary<br><br>(1:4000)	Santa Cruz Biotech	Cat# sc-2004 RRID:AB_631746
Anti-rat secondary<br><br>(1:4000)	Santa Cruz Biotech	Cat# sc-2006 RRID:AB_1125219
Compounds		
Ganetespib	Madrigal Pharma (formerly Synta Pharmaceuticals)	STA-9090 CAS# 888216-25-9<br><br>(Ying, Du et al., 2012)
Ganetespib, Biotinylated	Madrigal Pharma (formerly Synta Pharmaceuticals)	STA-12-7191
SNX-2112	Duke University<br><br>Dr. Timothy Haystead	CAS# 908112-43-6<br><br>(Barrott & Haystead, 2013)
Bortezomib	LC Labs	Cat# B-1408; CAS# 179324-69-7
JG-98	University of California at San Francisco<br><br>Dr. Jason Gestwicki	(Li, Srinivasan et al., 2013)
DPH	Sigma-Aldrich	Cat# SML0202-5MG CAS# 484049-04-9
GNF-5	Sigma-Aldrich	Cat# SML0511-5MG CAS# 778277-15-9
Critical Commercial Assays		
Mirus TransIT-2020	MirusBio	Cat# MIR5405
PiPer Phosphate Assay	ThermoFisher Scientific	Cat# P22061

Anti-FLAG M2 affinity gel	Sigma-Aldrich	Cat# A2220<br>RRID:AB10063035
Protein G agarose	ThermoFisher Scientific	Cat# 15-920-010
ATP agarose	Novus Biologicals	Cat# 510-0002
Strep agarose	ThermoFisher Scientific	Cat# 20349
Ni-NTA Agarose	ThermoFisher Scientific	Cat# 88221
Experimental Models: Cell Lines		
HEK293	ATCC Cat# CRL-1573	RRID:CVCL_0045
TSC1 (+/+) MEF	Brigham and Women's Hospital<br>Dr. David Kwiatkowski	(Kwiatkowski, Zhang et al., 2002)
TSC1 (–/–) MEF	Brigham and Women's Hospital<br>Dr. David Kwiatkowski	(Kwiatkowski et al., 2002)
c–Abl (+/+) MEF	Columbia University<br>Dr. Stephen Goff	(Suzuki, Sukezane et al., 2004)
c–Abl (–/–) MEF	Columbia University<br>Dr. Stephen Goff	(Suzuki et al., 2004)
Experimental Models: Organisms/Strains		
PP30-Hsp90α	University of Sheffield<br>Dr. Peter Piper	(Piper, Millson et al., 2003)
PP30-Hsp82	University of Sheffield<br>Dr. Peter Piper	(Panaretou, Prodromou et al., 1998)
Oligonucleotides		

Tsc1 siRNA	GE Dharmacon<br><br>Cat# L-003028-00-0005	1-CGACACGGCUGAUAACUGA<br><br>2-CGGCUGAUGUUGUUAAAUA<br><br>3-GUUACUGGGUCAUGUCAUA<br><br>4-CAAAGCCGCCACUCGUUUA
Non-targeting siRNA	GE Dharmacon<br><br>Cat# D-001810-10-20	1-UGGUUUACAUGUCGACUAA<br><br>2-UGGUUUACAUGUUGUGUGA<br><br>3-UGGUUUACAUGUUUUCUGA<br><br>4-UGGUUUACAUGUUUUCCUA

Primer Name	Primer Sequence
Tsc1-A-KpnI-FLAG-F	TATGCGGGTACCATGGATTACAAGGATGACGACGATAAGGGAGCCCAACAAGCAAATGTCGGGGAGCTTC
Tsc1-A-ApaI-R	TATGCGGGGCCCTTAATTCTGTGTGTCAGCATAAGGGC
Tsc1-B-KpnI-FLAG-F	TATGCGGGTACCATGGATTACAAGGATGACGACGATAAGGGAAGCTATGGGTGTGCTACTTC
Tsc1-B-ApaI-R	TATGCGGGGCCCTTATGGTCTTGCAGAATCCATTC
Tsc1-C-KpnI-FLAG-F	TATGCG GGTACCATGGATTACAAGGATGACGACGATAAGGGATGTCTACACAGACAACACC
Tsc1-C-ApaI-R	TATGCG GGGCCCTTAGTCATTACAACAGTCAAGCCT
Tsc1-D-KpnI-FLAG-F	TATGCGGGTACCATGGATTACAAGGATGACGACGATAAGGGAGACGGGTGCTCAGATTC
Tsc1-D-ApaI-R	TATGCGGGGCCCTTAGCTGTGTTCATGATGAGTC
Tsc1-D-HA-KpnI-F	TATGCGGGTACCATGTACCCATACGATGTTCCAGATTACGCTGGGGACGGGTGCTCAGATTCC
Tsc1-D-NotI-R	TATGCGGCGGCCGCTTAGCTGTGTTCATGATGAGTC

Hsp90a-TPR-BamHI-F	TATGCGGGATCCATGGATTACAAGGATGACGACGATAAGGGACCTGAGGAAACCCAGAC	
Hsp90a-TPR-XhoI-R	TATGCGCTCGAGTTAGCGTGATGTGTCGTCATC	
Hsp90B-TPR-NotI-F	TATGCGGCGGCCGCATGGATTACAAGGATGACGACGATAAGGGACCTGAGGAAGTGCAC	
Hsp90B-TPR-XbaI-R	TATGCGTCTAGATTAGCGAGACGCATCCTCATC	
Hsp90-V410E-F	AGCAAAATTTTGAAAGAAATCAGGAAGAATTTG	
Hsp90-V410E-R	CAAATTCTTCCTGATTTCTTTCAAAATTTTGCT	
Aha1-F-Spe1	TATGCACTAGTATGGCCAAGTGGGGTGAGGGA	
Aha1-R-Xho1	TCGGTCTCGAGTTACTTGTCATCGTCGTCCTTGTAGTCAAATAAGCGTGCGCCATAGCCAAAGGTCTGT	
Tsc1-L117P-F	TTGCCTTCTTTACCAAAATGTCTCAAG	
Tsc1-L117P-R	CTTGAGACATTTTGGTAAAGAAGGCAA	
Recombinant DNA		
pcDNA3-FLAG-TSC2	Addgene Cat# 14129	(Tee, Fingar et al., 2002)
pcDNA3-HA3-TSC1	Addgene Cat# 19911	(Tee et al., 2002)
pcDNA3-cFLAG	Addgene Cat# 20011	(Sanjabi, Williams et al., 2005)
pcDNA3.1-CFTR	University of Pittsburgh<br><br>Dr. Brodsky	(Ahner, Whyte et al., 2005)
p424-ADH	ATCC	Cat# 87373
pYES2	ThermoFisher Scientific	Cat# V82520

Appendix References

Ahner A, Whyte FM, Brodsky JL (2005) Distinct but overlapping functions of Hsp70, Hsp90, and an Hsp70 nucleotide exchange factor during protein biogenesis in yeast. Arch Biochem Biophys 435: 32-41

Barrott JJ, Haystead TA (2013) Hsp90, an unlikely ally in the war on cancer. FEBS J 280: 1381-96

Kwiatkowski DJ, Zhang H, Bandura JL, Heiberger KM, Glogauer M, el-Hashemite N, Onda H (2002) A mouse model of TSC1 reveals sex-dependent lethality from liver hemangiomas, and up-regulation of p70S6 kinase activity in Tsc1 null cells. Hum Mol Genet 11: 525-34

Li X, Srinivasan SR, Connarn J, Ahmad A, Young ZT, Kabza AM, Zuiderweg ER, Sun D, Gestwicki JE (2013) Analogs of the Allosteric Heat Shock Protein 70 (Hsp70) Inhibitor, MKT-077, as Anti-Cancer Agents. ACS Med Chem Lett 4

Panaretou B, Prodromou C, Roe SM, O'Brien R, Ladbury JE, Piper PW, Pearl LH (1998) ATP binding and hydrolysis are essential to the function of the Hsp90 molecular chaperone in vivo. EMBO J 17: 4829-36

Piper PW, Millson SH, Mollapour M, Panaretou B, Siligardi G, Pearl LH, Prodromou C (2003) Sensitivity to Hsp90-targeting drugs can arise with mutation to the Hsp90 chaperone, cochaperones and plasma membrane ATP binding cassette transporters of yeast. Eur J Biochem 270: 4689-95

Sanjabi S, Williams KJ, Saccani S, Zhou L, Hoffmann A, Ghosh G, Gerondakis S, Natoli G, Smale ST (2005) A c-Rel subdomain responsible for enhanced DNA-binding affinity and selective gene activation. Genes Dev 19: 2138-51

Suzuki J, Sukezane T, Akagi T, Georgescu MM, Ohtani M, Inoue H, Jat PS, Goff SP, Hanafusa H, Shishido T (2004) Loss of c-abl facilitates anchorage-independent growth of p53- and RB- deficient primary mouse embryonic fibroblasts. Oncogene 23: 8527-34

Tee AR, Fingar DC, Manning BD, Kwiatkowski DJ, Cantley LC, Blenis J (2002) Tuberous sclerosis complex-1 and -2 gene products function together to inhibit mammalian target of rapamycin (mTOR)-mediated downstream signaling. Proc Natl Acad Sci U S A 99: 13571-6

Ying W, Du Z, Sun L, Foley KP, Proia DA, Blackman RK, Zhou D, Inoue T, Tatsuta N, Sang J, Ye S, Acquaviva J, Ogawa LS, Wada Y, Barsoum J, Koya K (2012) Ganetespib, a unique triazolone-containing Hsp90 inhibitor, exhibits potent antitumor activity and a superior safety profile for cancer therapy. Mol Cancer Ther 11: 475-84
