# Supplementary material for: Tumor suppressor Tsc1 is a new Hsp90 co‐chaperone that facilitates folding of kinase and non‐kinase clients
Source: EMBO J. 2017 Nov 10;36(24):3650–65. doi: 10.15252/embj.201796700 (PMC5730846; doi:10.15252/embj.201796700)
Supplement: Supplementary file 2 — Expanded View Figures PDF [file EMBJ-36-3650-s002.pdf]

Expanded View Figures

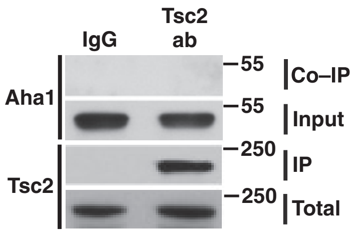

Figure EV1. Tsc2 interaction with the chaperone and co-chaperone machinery (related to Fig 1).

Tsc2 was immunoprecipitated from HEK293 cell lysates using anti-Tsc2 antibody and immunoblotted with indicated antibodies. IgG was used as control. These samples are the same as those presented in Fig 1A.

Source data are available online for this figure.

Figure EV2. Inhibition of the Hsp90 function by the co-chaperone Tsc1 (related to Fig 2).

A Tsc1 was immunoprecipitated from HEK293 cell lysates using anti-Tsc1 antibody and immunoblotted with indicated antibodies. IgG was used as control. These samples are the same as those presented in Fig 2A.

B BSA binding affinity to bacterially expressed and purified Hsp90α was determined by fluorescence anisotropy. The error bars represent mean ± SD of three independent experiments.

C Inorganic phosphate (P_i) standard curve. The *x*-axis shows μM of P_i per assay and the *y*-axis shows absorbance at 565 nm. Mean ± SD from values obtained in three independent experiments.

D ATPase activity of Hsp90α *in vitro*. Inhibitory effects of purified Tsc1-D-His_6 on the ATPase activity of Hsp90α are shown. All the data represent mean ± SD of three biological replicates. A Student's *t*-test was performed to assess statistical significance (**$P < 0.005$, ****$P < 0.0001$).

E Different amounts of BSA have no effect on Hsp90 ATPase activity *in vitro*. All the data represent mean ± SD of three biological replicates.

F ATPase activity of Hsp90 from five *Tsc1*^{GFAP}CKO (K1–K5) mice with conditional inactivation of the *TSC1* gene in glia and five *Tsc1*$^{flox/+}$-GFAP-Cre and *Tsc1*$^{flox/flox}$ littermate controls (C1–C5). All the data represent mean ± SD of three biological replicates. A Student's *t*-test was performed to assess statistical significance (**$P < 0.005$); 50 ng of the isolated Hsp90 was resolved on the SDS–PAGE gel and stained with Coomassie stain.

Source data are available online for this figure.

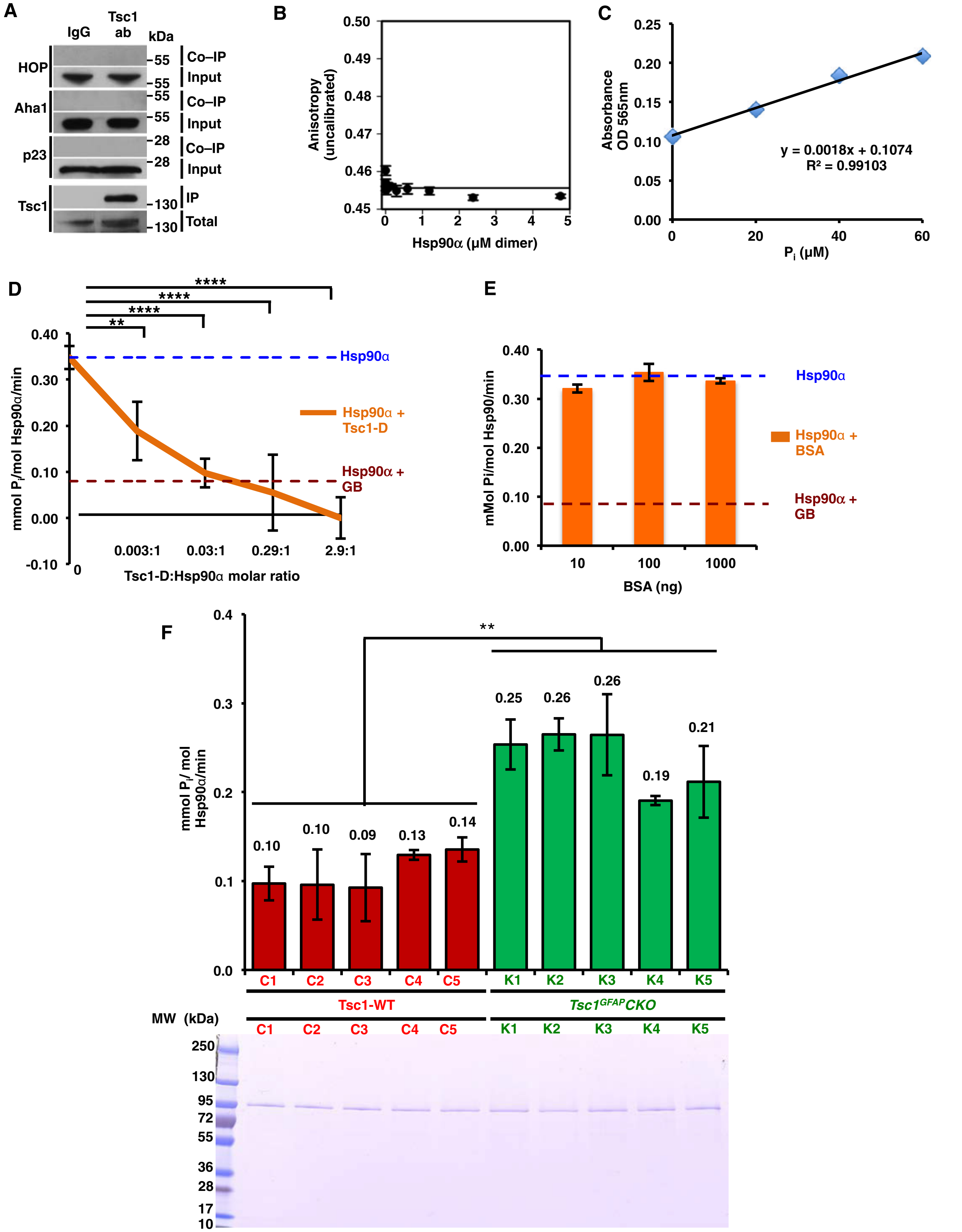

Figure EV2.

 © 2017 The Authors

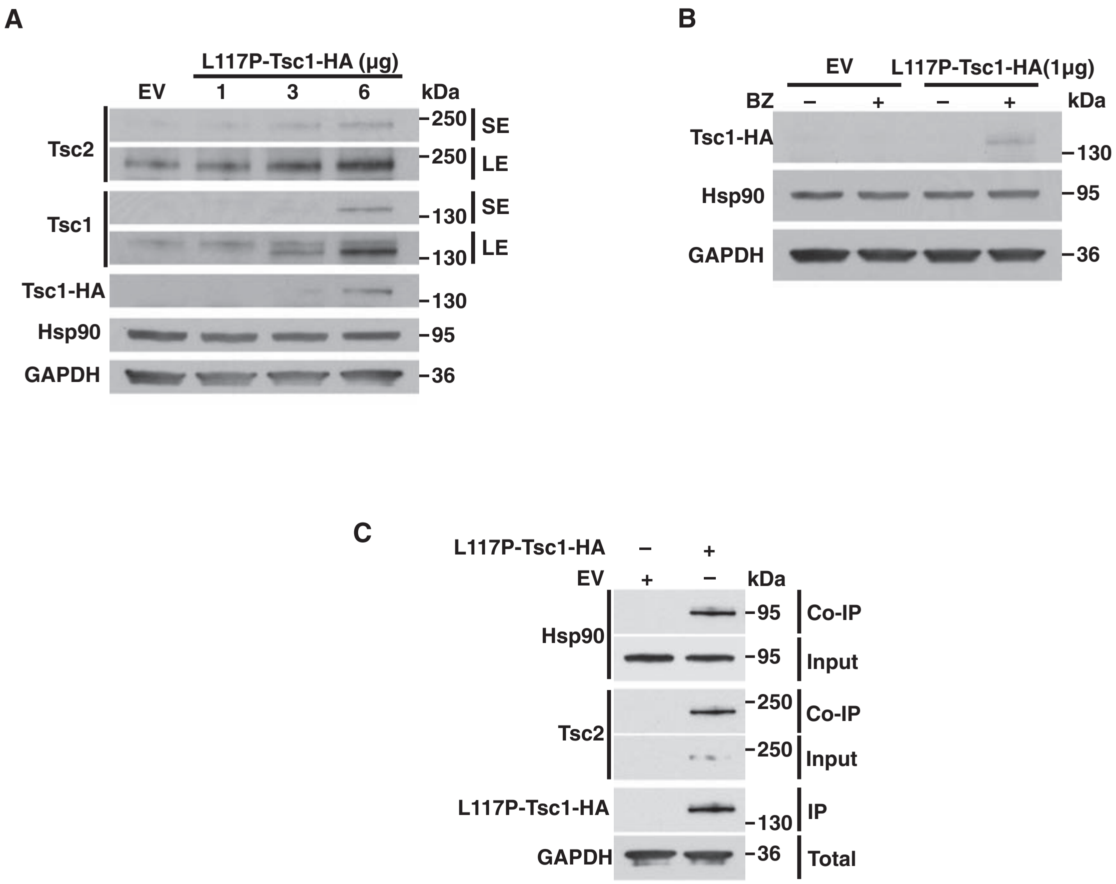

Figure EV3. Expression and interaction of the pathogenic mutant L117P-Tsc1 with Hsp90 and Tsc2 (related to Fig 3).

A Tsc1$^{-/-}$ MEF cells were transiently transfected with indicated amounts of L117P-Tsc1-HA and detected by immunoblotting. SE (short exposure) and LE (long exposure) of the radiographic film.

B Tsc1$^{-/-}$ MEF cells transiently expressing either 1 μg of empty vector or L117P-Tsc1-HA plasmid for 24 h, followed by 50 nM proteasome inhibitor BZ treatment for 2 h. Samples were analyzed by immunoblotting.

C 6 μg of empty vector or L117P-Tsc1-HA plasmid for was transiently expressed in Tsc1$^{-/-}$ MEF cells, and L117P-Tsc1-HA was immunoprecipitated and co-immunoprecipitation of Hsp90 and Tsc2 was analyzed by immunoblotting.

Source data are available online for this figure.

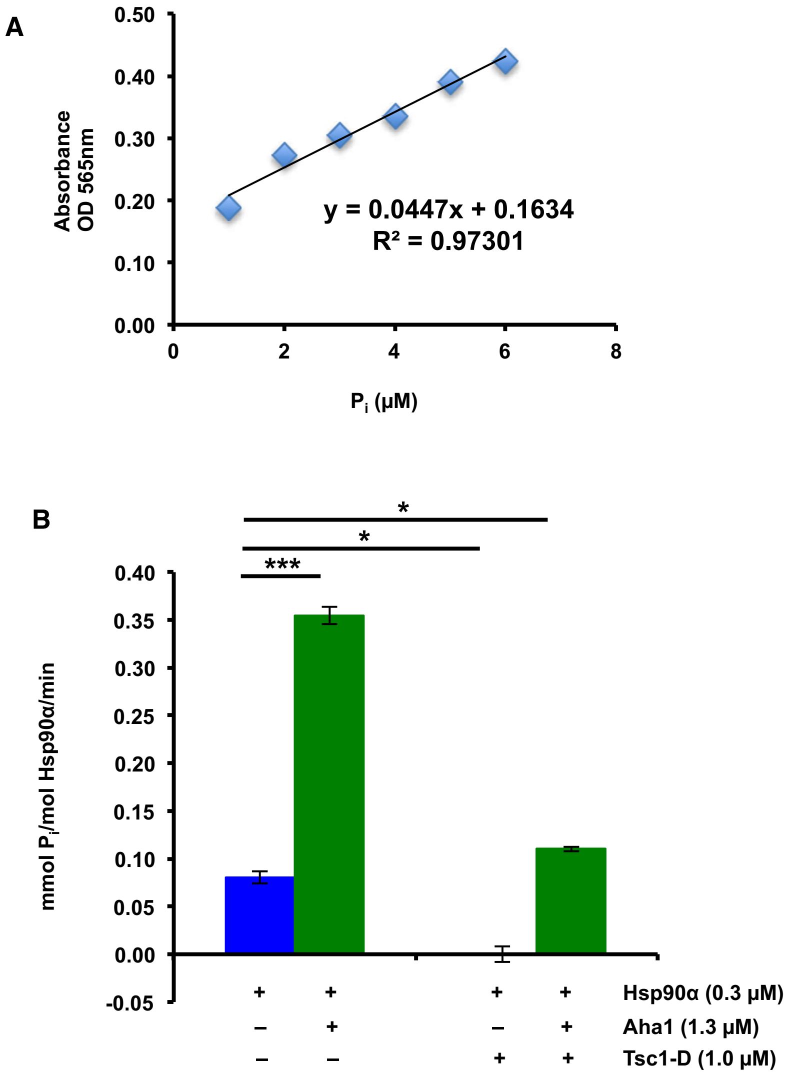

Figure EV4. Tsc1 and Aha1 compete for binding and regulating Hsp90 function (related to Fig 5).

A Inorganic phosphate (P_i) standard curve. The *x*-axis shows μM of P_i per assay and the *y*-axis shows absorbance at 565 nm. Mean ± SD from values obtained in three independent experiments.

B Tsc1-D-His_6 inhibits Hsp90α ATPase activity after 30 min. Addition of 1.3 μM Aha1-FLAG stimulated the ATPase activity. All the data represent mean ± SD of three biological replicates. A Student's *t*-test was performed to assess statistical significance (*$P < 0.05$, ***$P < 0.0005$).

 © 2017 The Authors
