# Supplementary figures and images for: Tumor suppressor Tsc1 is a new Hsp90 co‐chaperone that facilitates folding of kinase and non‐kinase clients
Source: EMBO J. 2017 Nov 10;36(24):3650–65. doi: 10.15252/embj.201796700 (PMC5730846; doi:10.15252/embj.201796700)

# Source Data Fig EV1

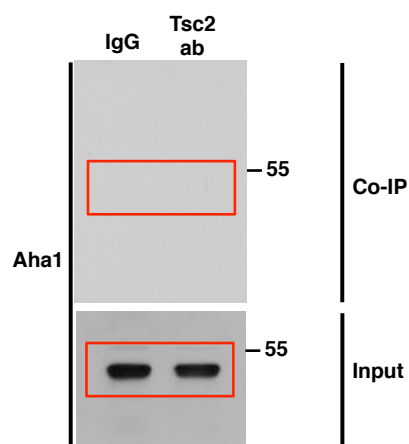

Tsc2 input and IP can be found in the Source Data for Fig 1A.

Supplement: Supplementary file 3 — Source Data for Expanded View [file EMBJ-36-3650-s009.zip › EMBOJ_96700_Sourcedata_fig_EV1.pdf]

# Source Data Fig EV2

**A**

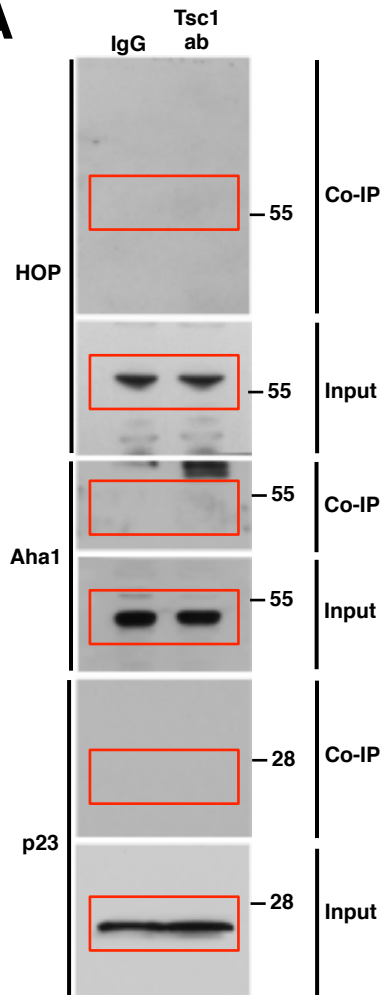

Tsc1 input and IP can be found in the Source Data for Fig 2A.

Supplement: Supplementary file 3 — Source Data for Expanded View [file EMBJ-36-3650-s009.zip › EMBOJ_96700_Sourcedata_fig_EV2.pdf]

# Source Data Fig EV3

A

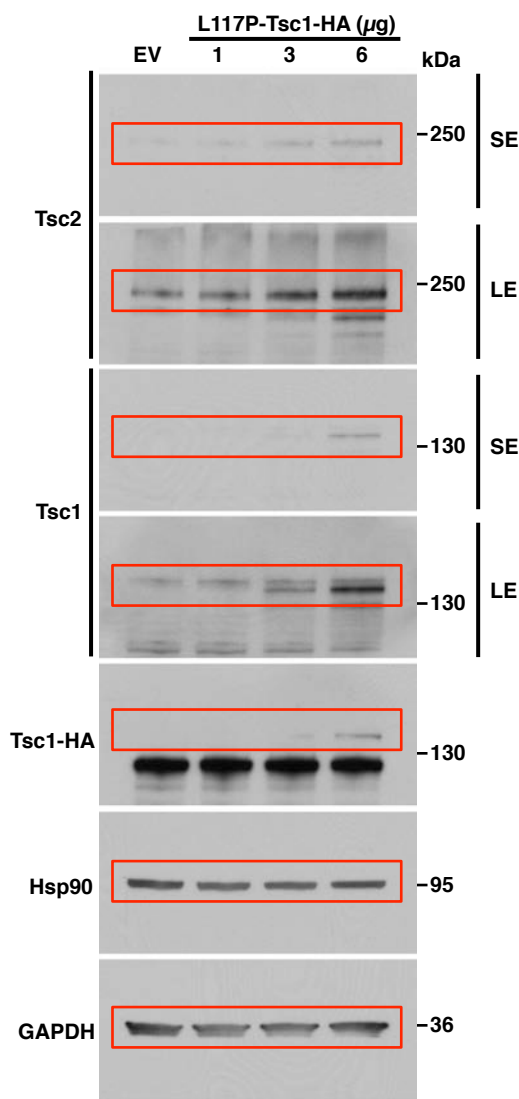

B

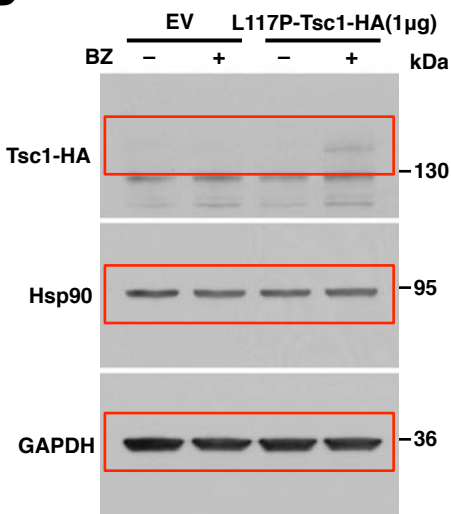

C

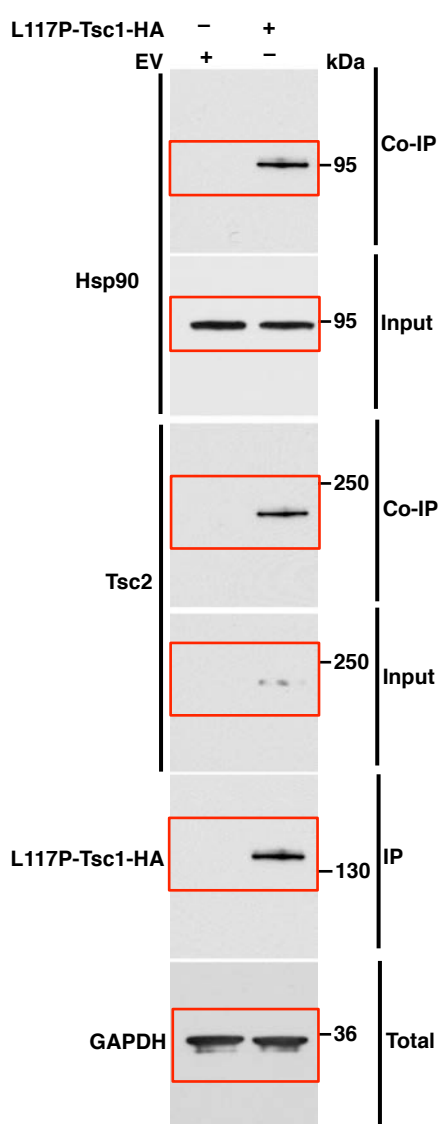

Supplement: Supplementary file 3 — Source Data for Expanded View [file EMBJ-36-3650-s009.zip › EMBOJ_96700_Sourcedata_fig_EV3.pdf]

Source Data Fig 1

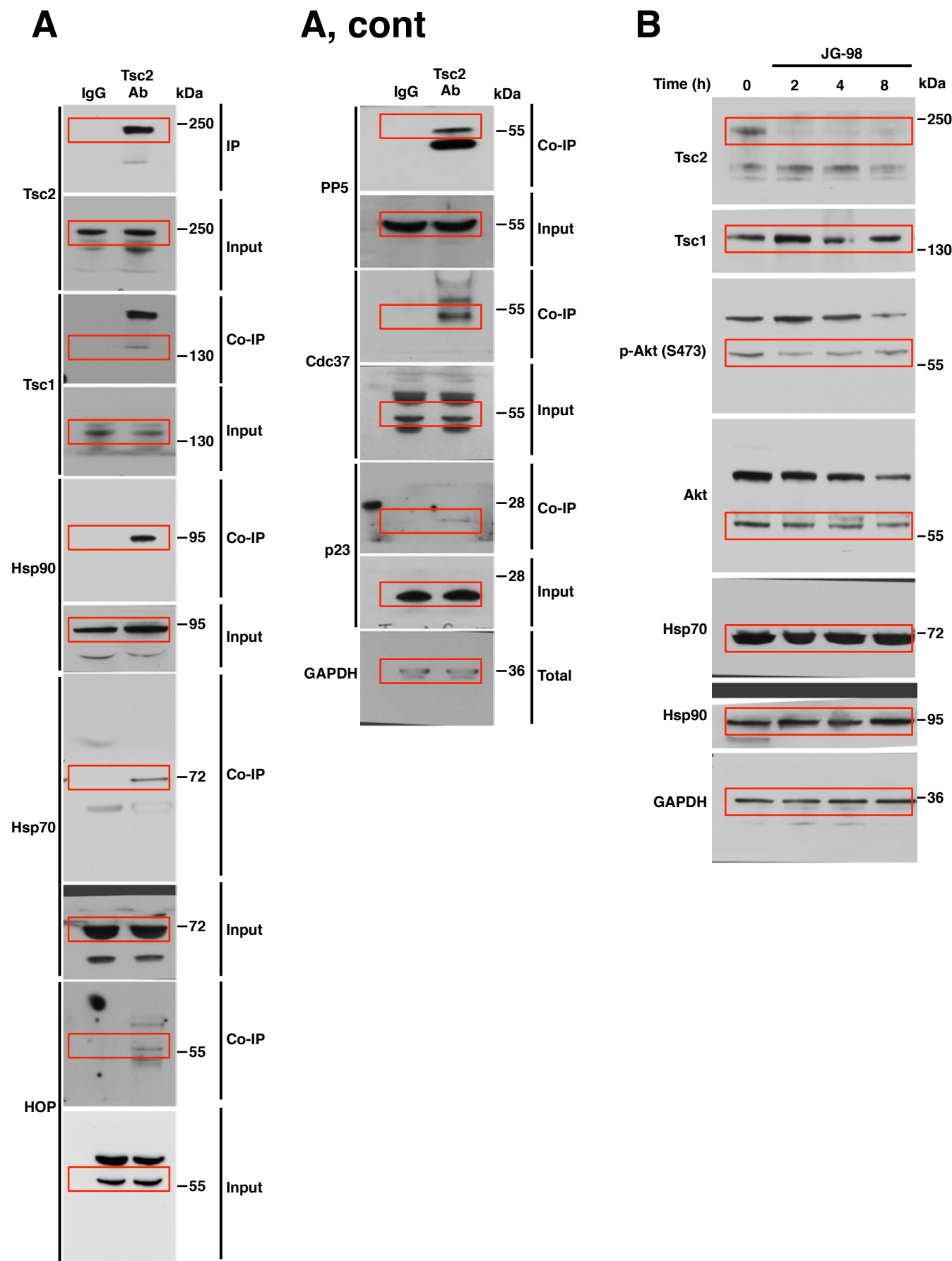

# Source Data Fig 1

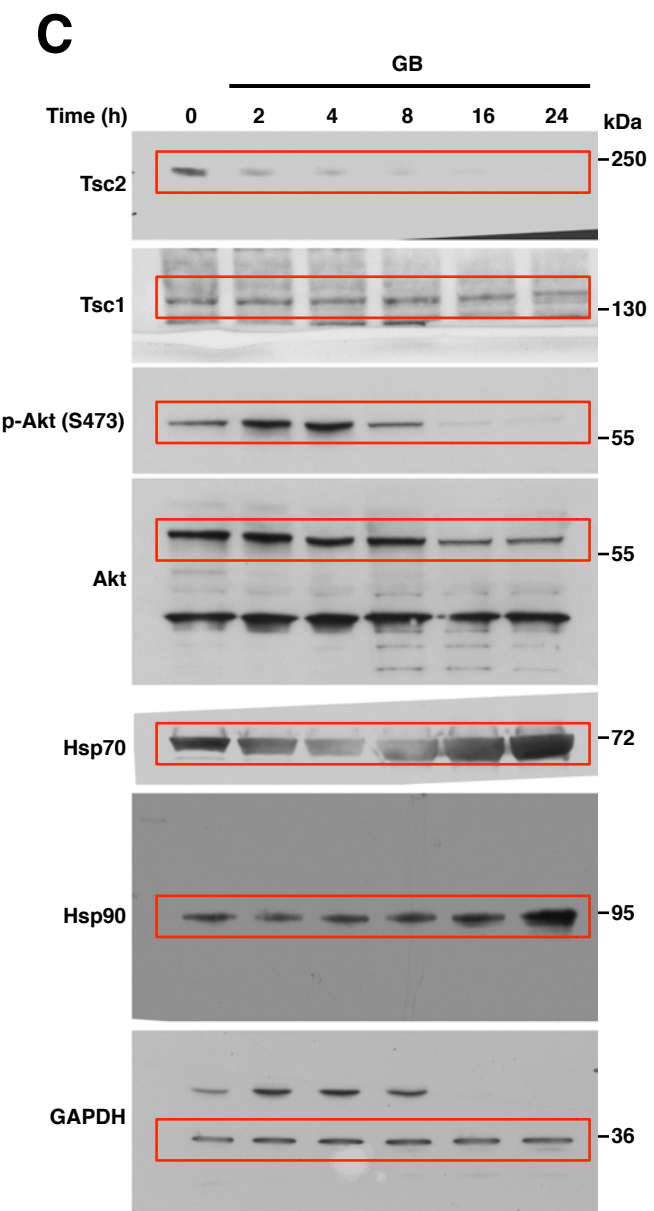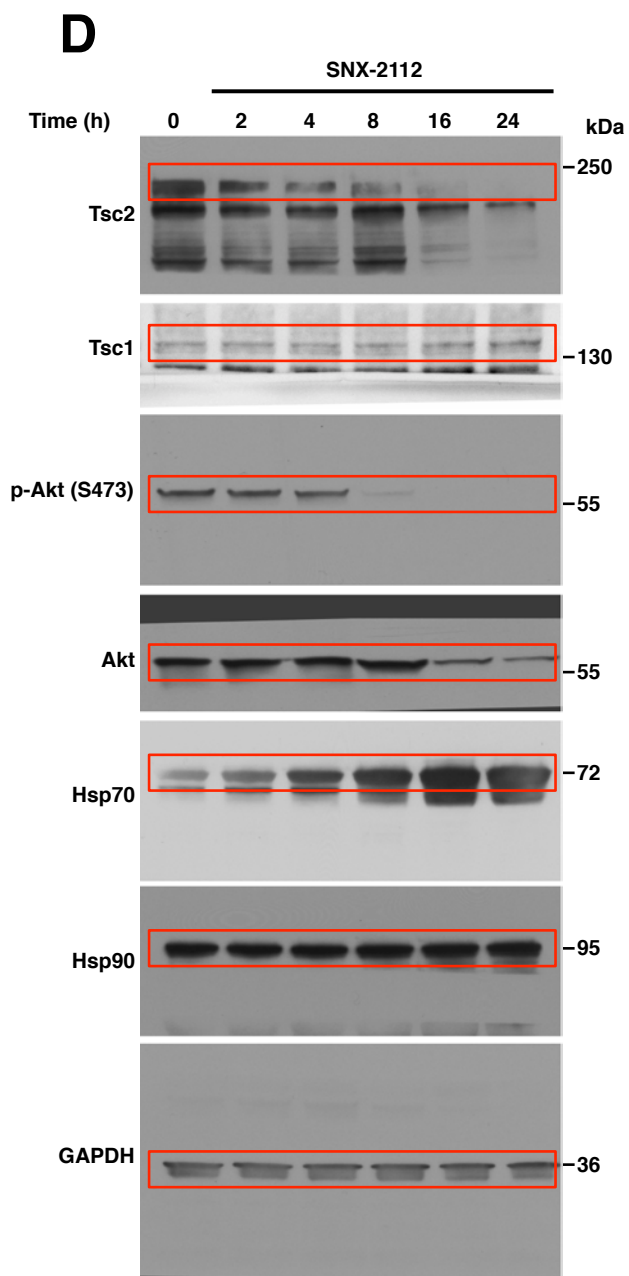

# Source Data Fig 1

E

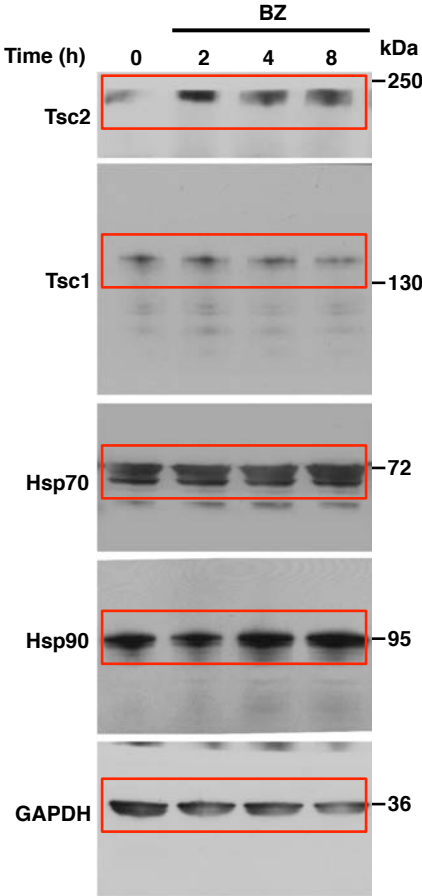

F

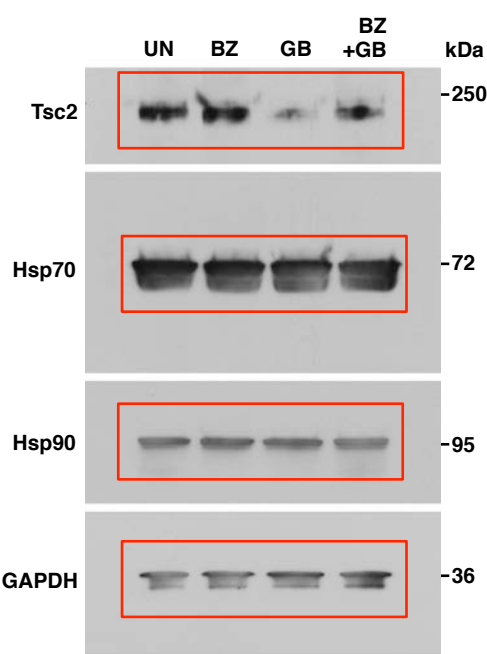

G

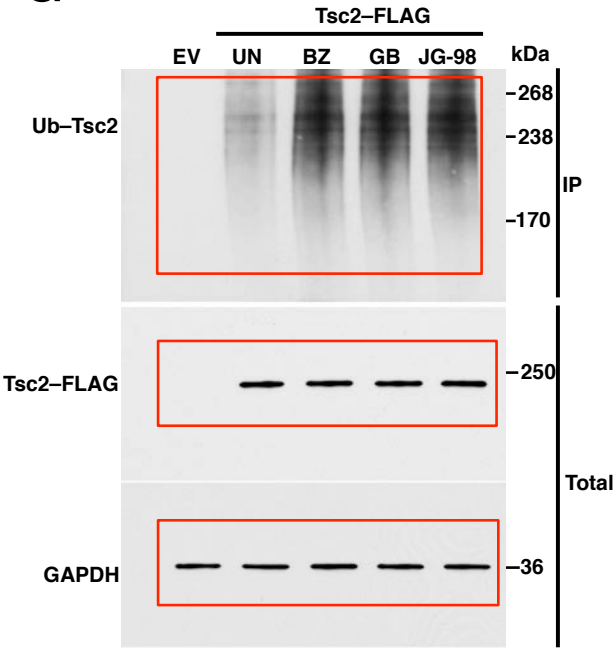

Supplement: Supplementary file 5 — Source Data for Figure 1 [file EMBJ-36-3650-s003.pdf]

Source Data Fig 2

A

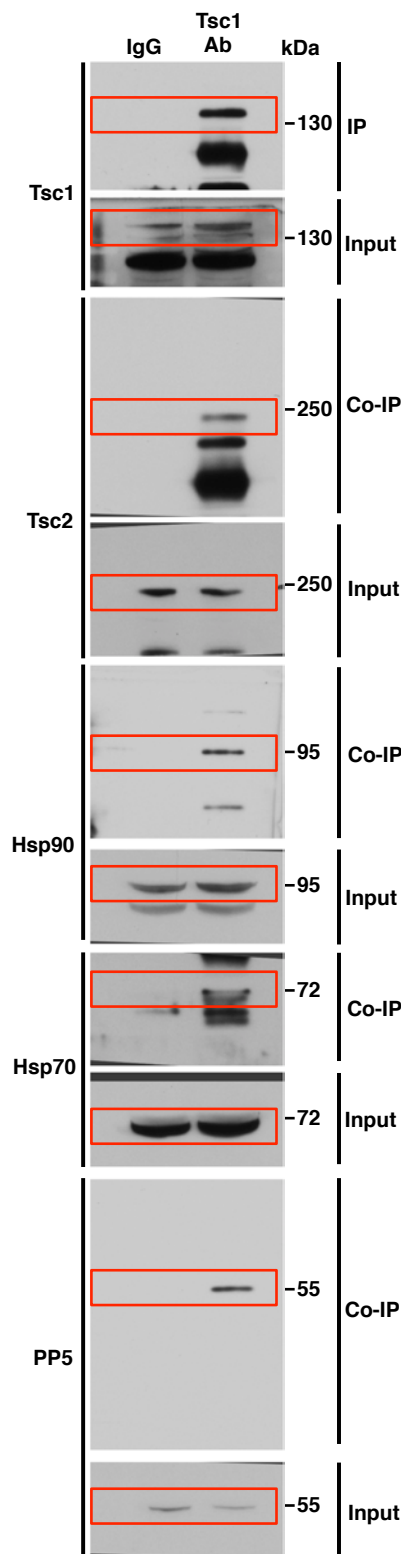

A, cont.

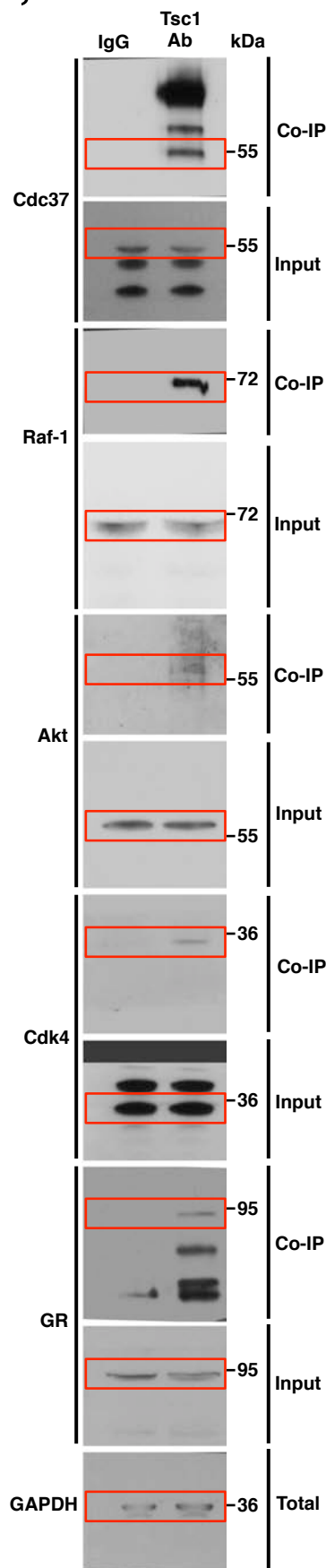

B

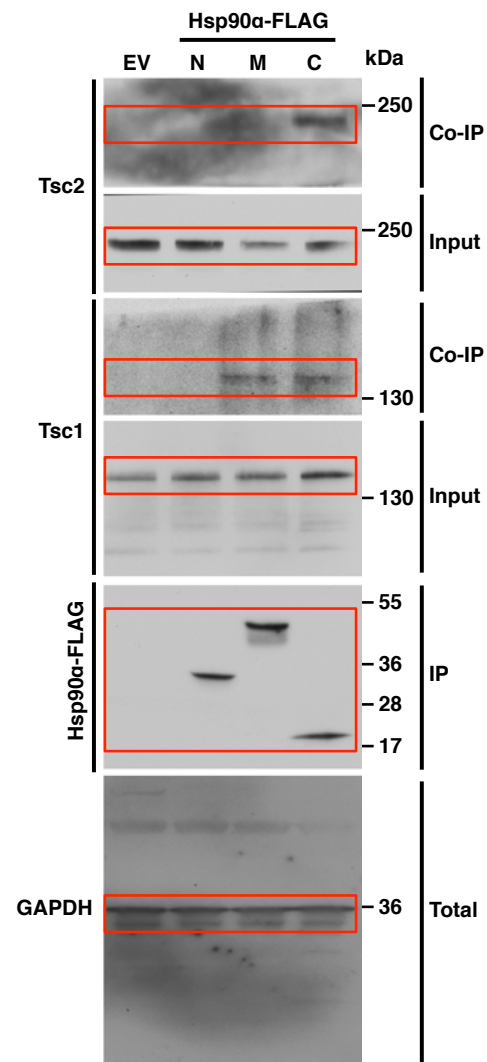

Source Data Fig 2

C

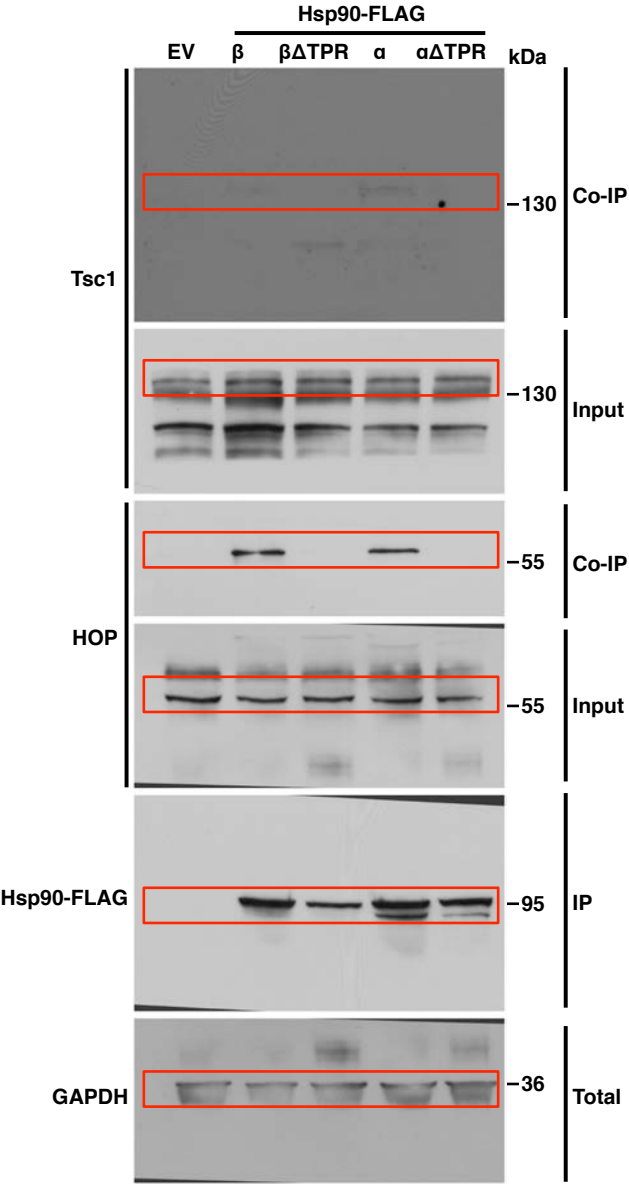

D

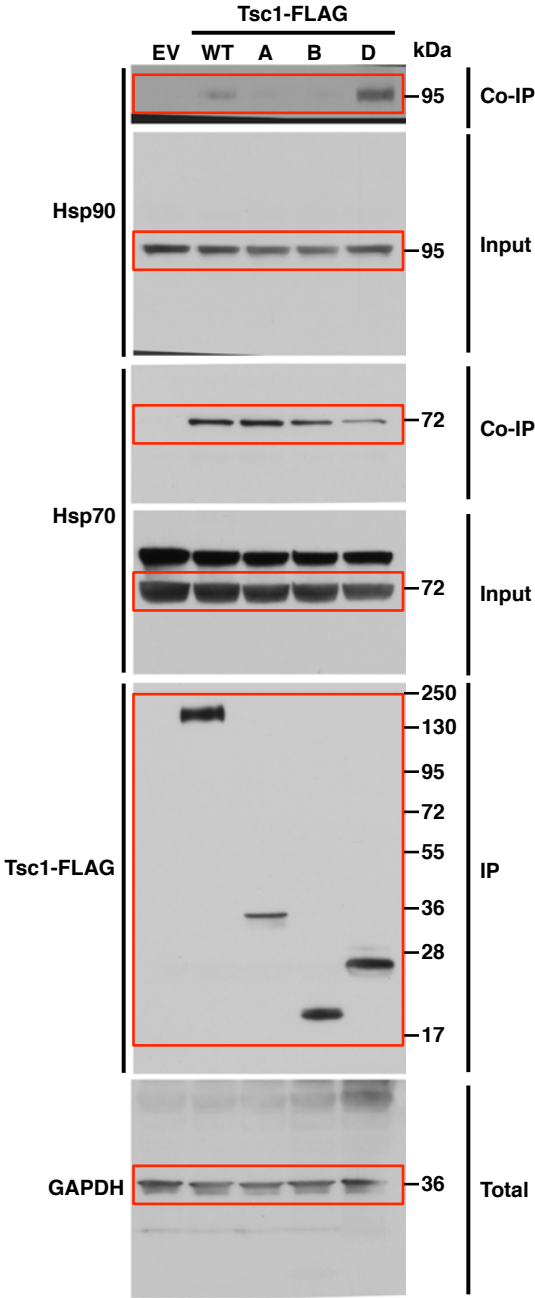

Source Data Fig 2

E

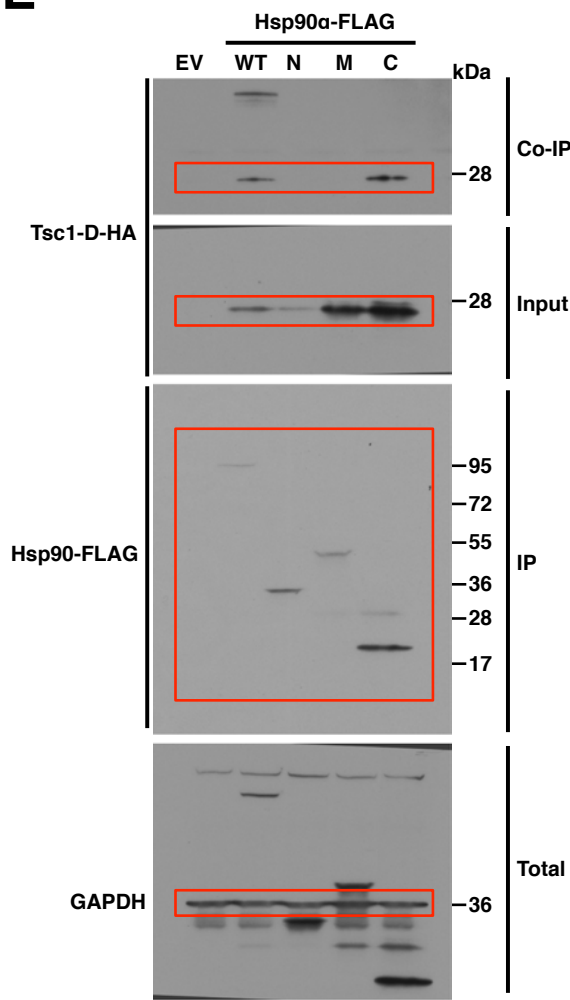

I

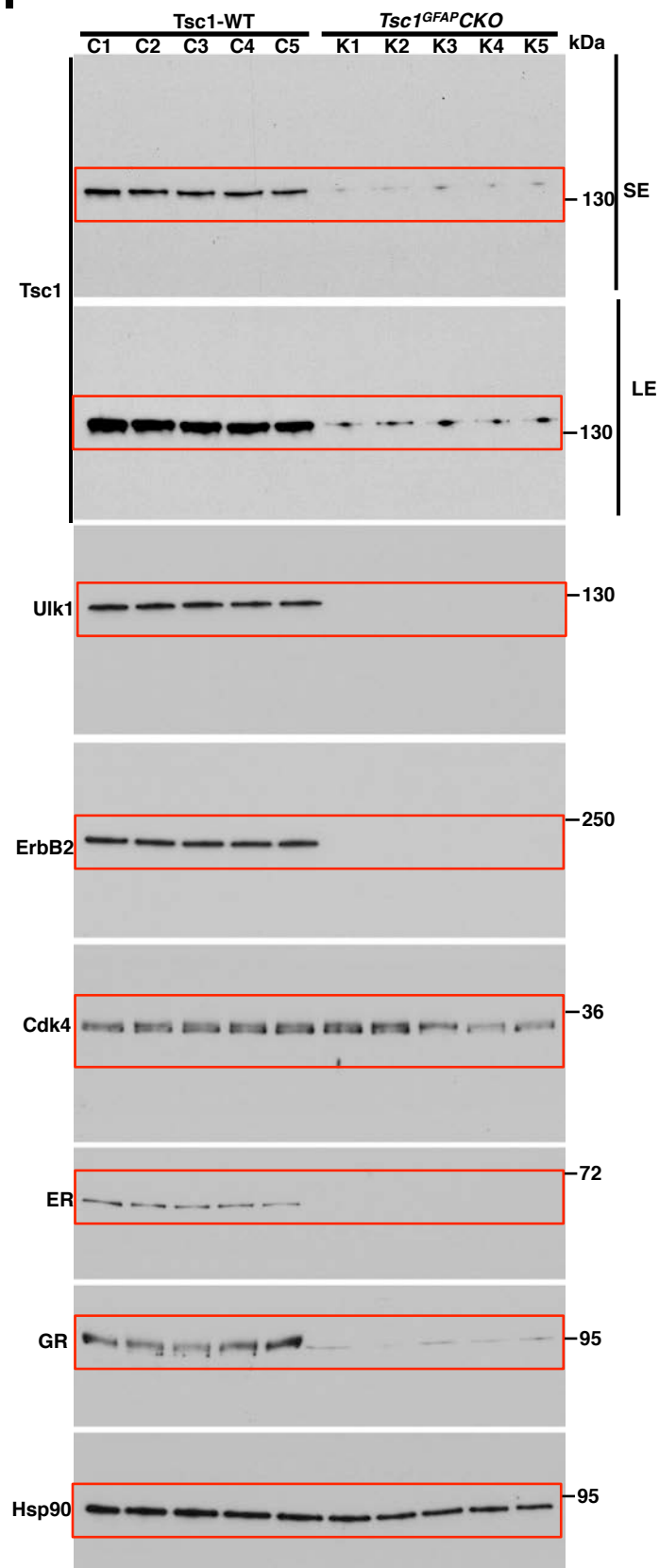

Supplement: Supplementary file 6 — Source Data for Figure 2 [file EMBJ-36-3650-s004.pdf]

Source Data Fig 3

A

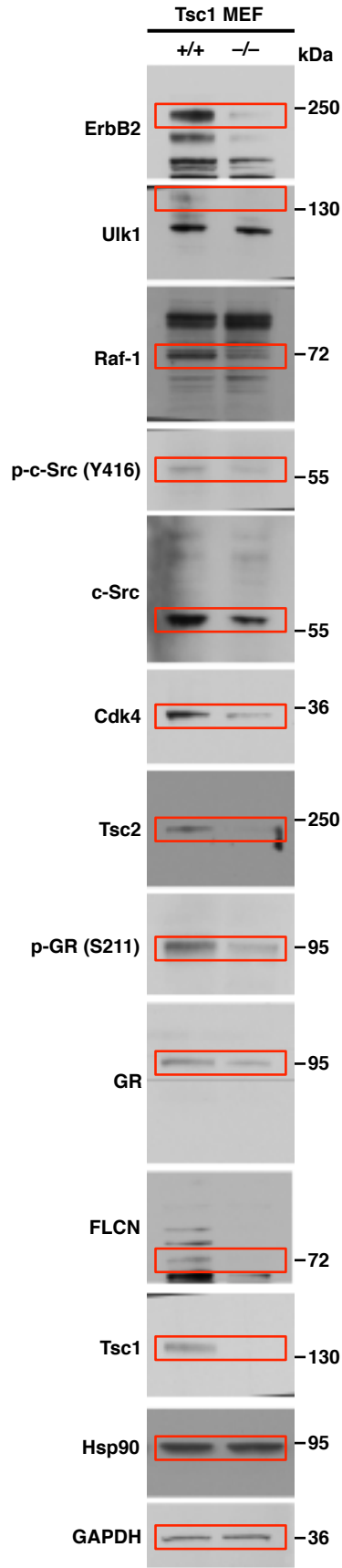

B

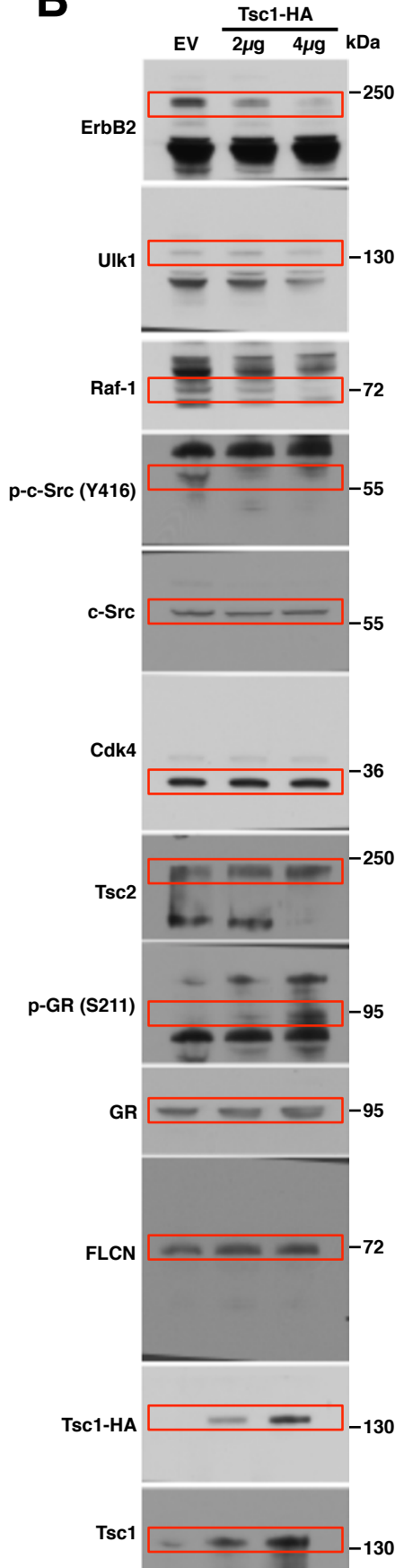

B, cont.

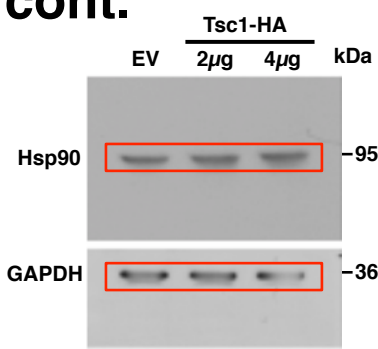

C

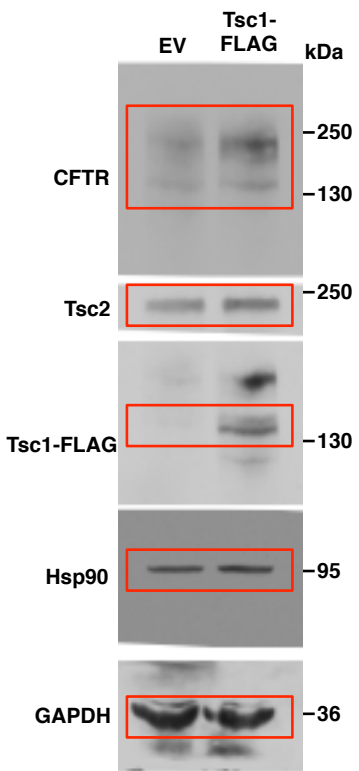

# Source Data Fig 3

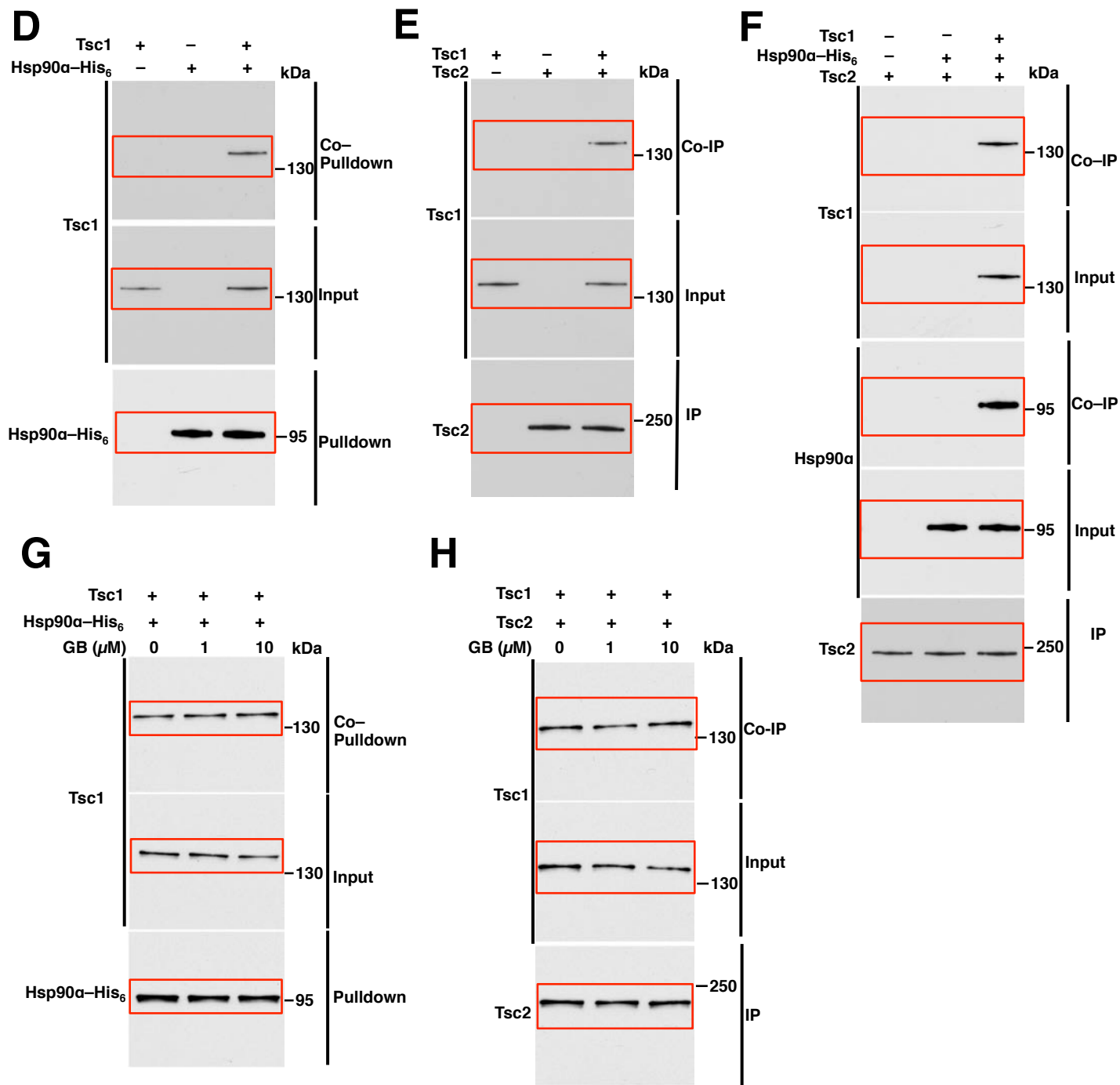

# Source Data Fig 3

I

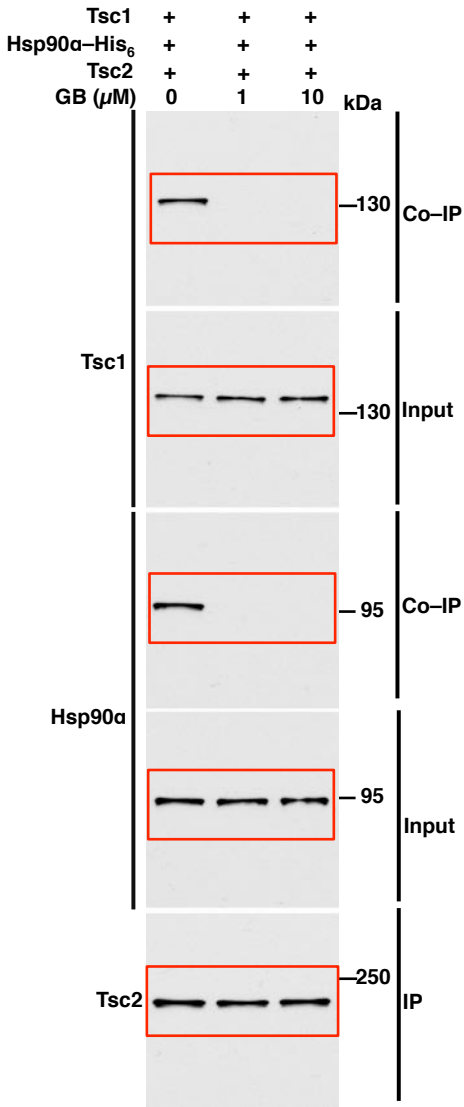

J

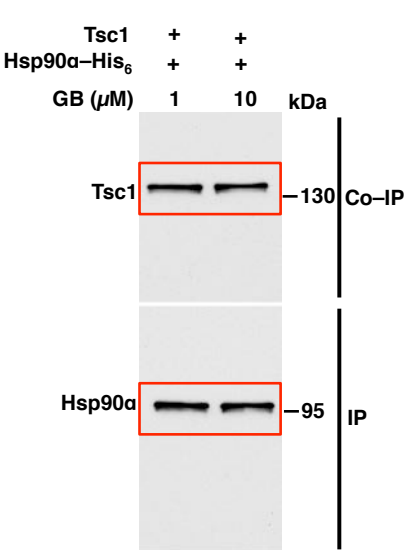

Supplement: Supplementary file 7 — Source Data for Figure 3 [file EMBJ-36-3650-s005.pdf]

# Source Data Fig 4

A

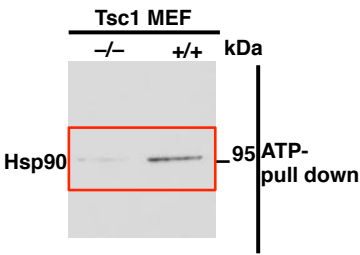

B

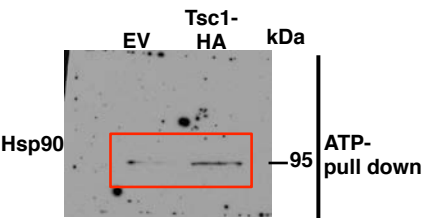

C

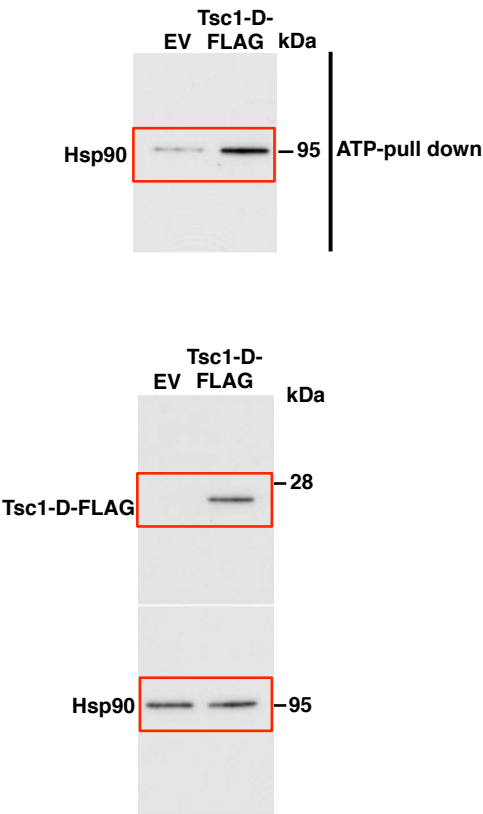

D

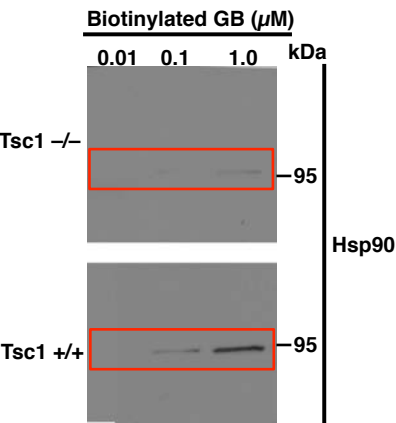

E

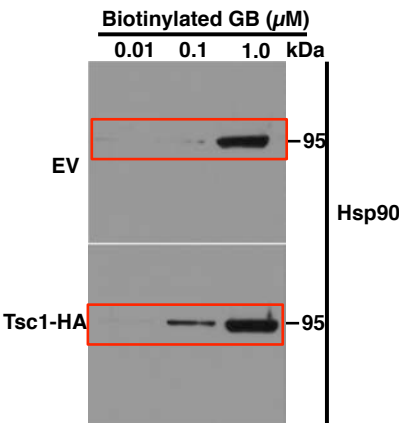

F

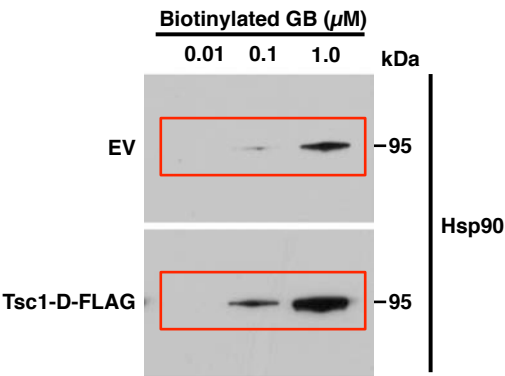

Source Data Fig 4

G

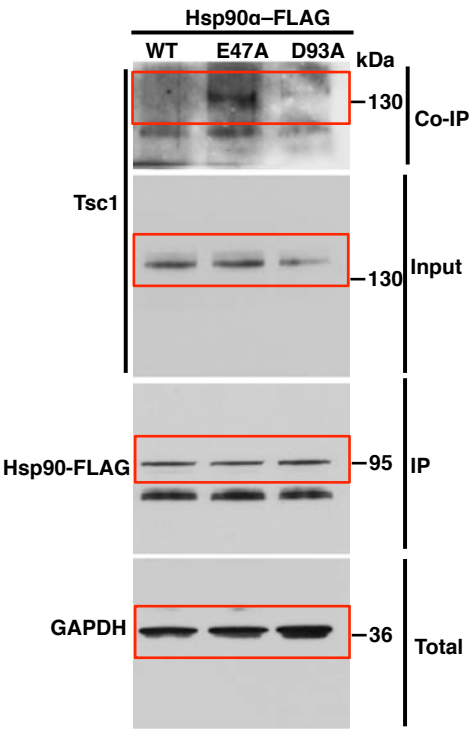

H

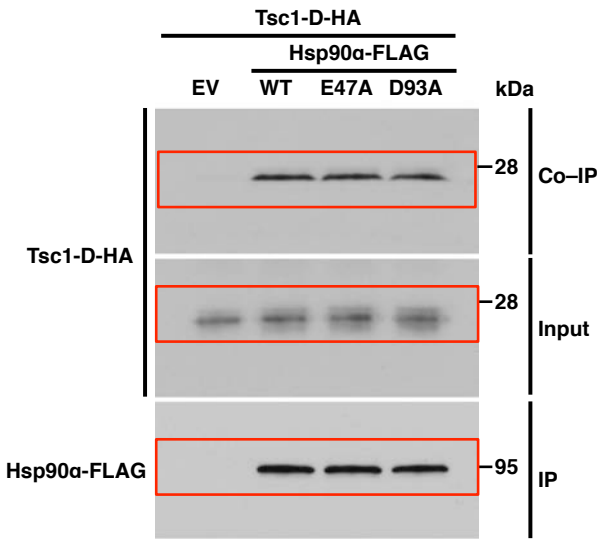

Supplement: Supplementary file 8 — Source Data for Figure 4 [file EMBJ-36-3650-s006.pdf]

Source Data Fig 5

A

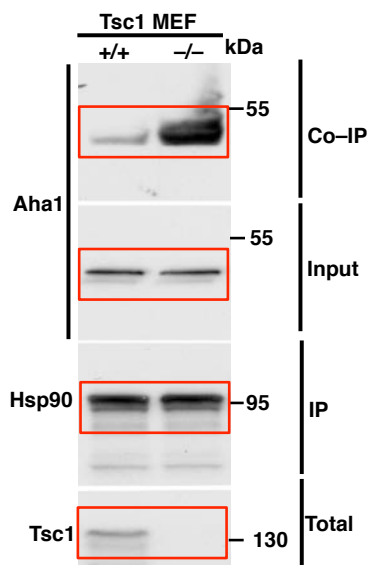

C

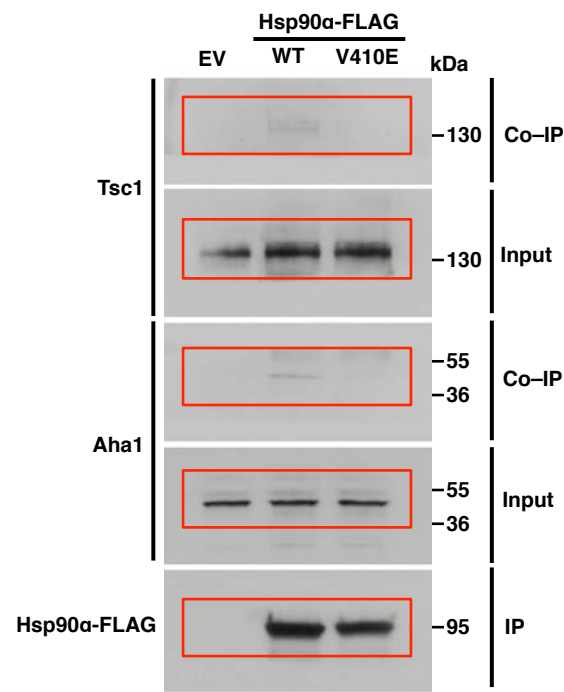

B

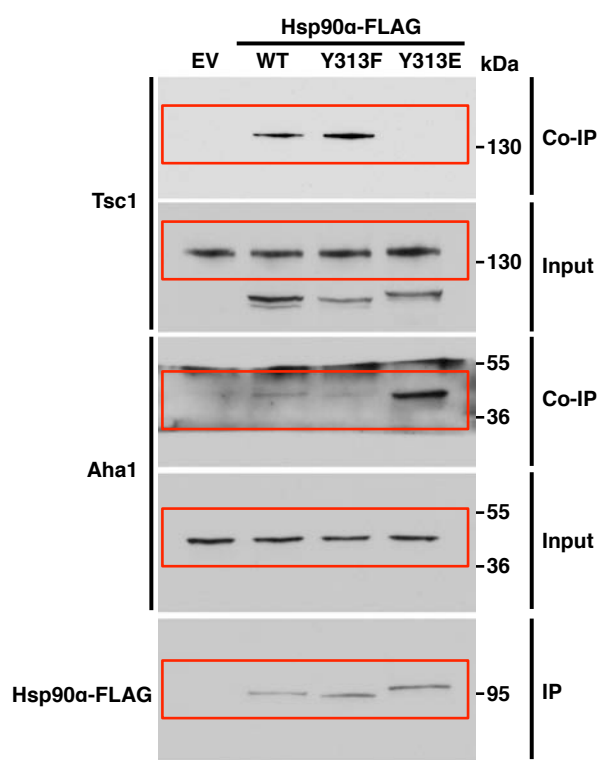

D

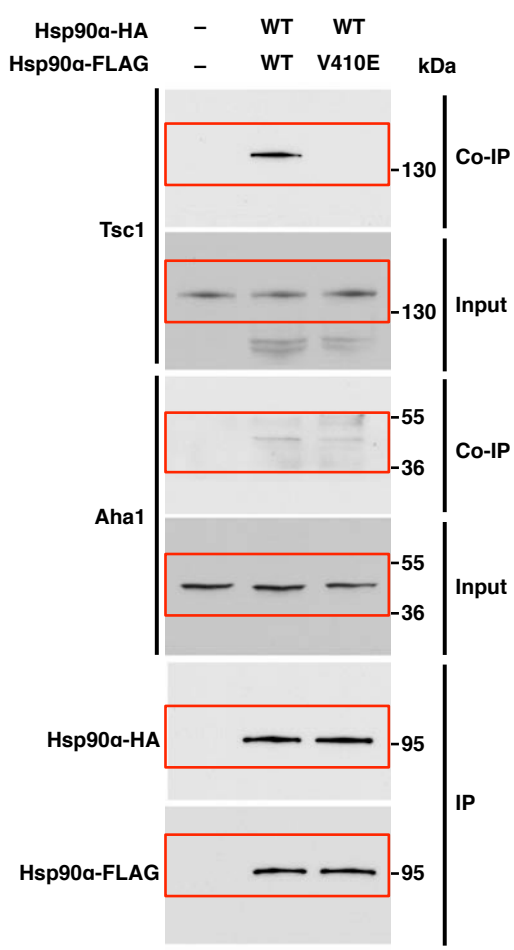

# Source Data Fig 5

E

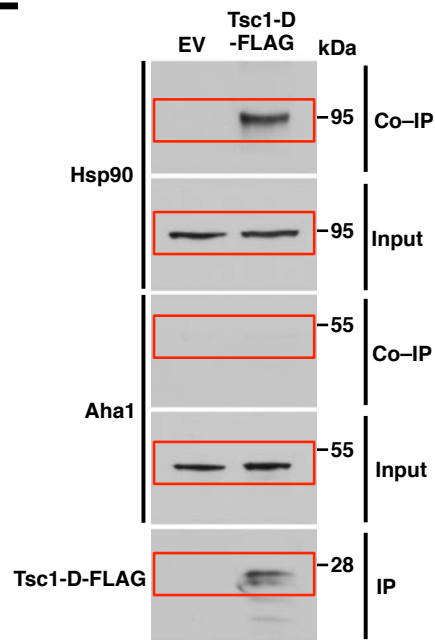

H

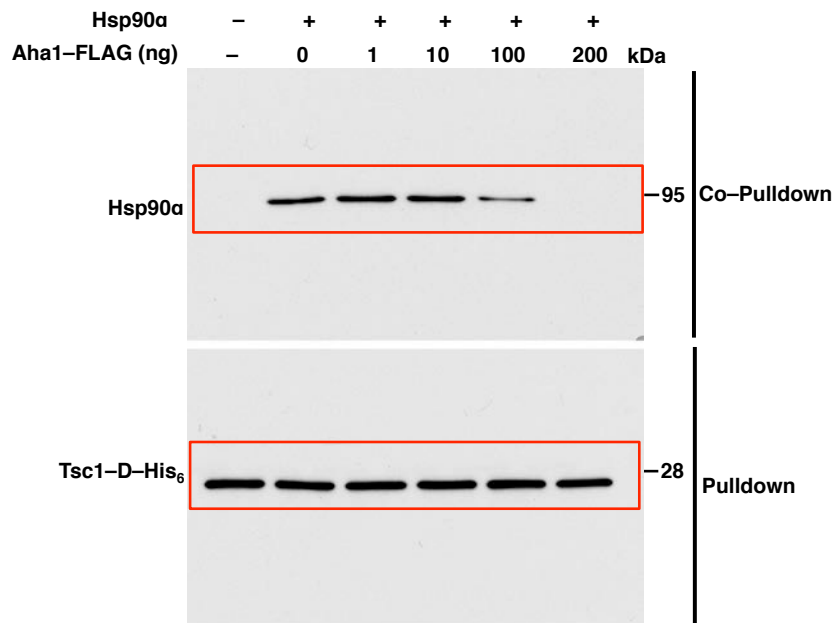

F

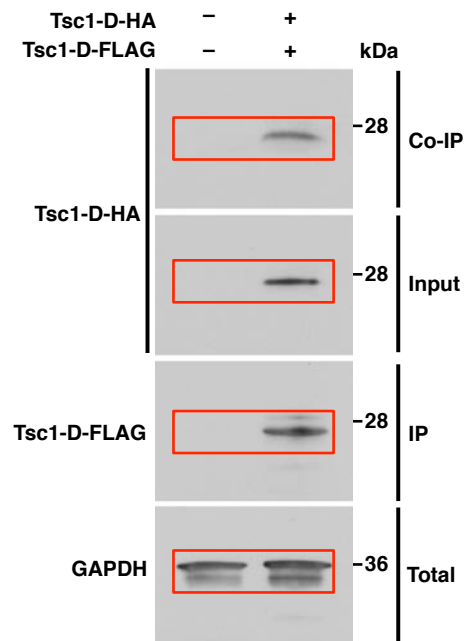

I

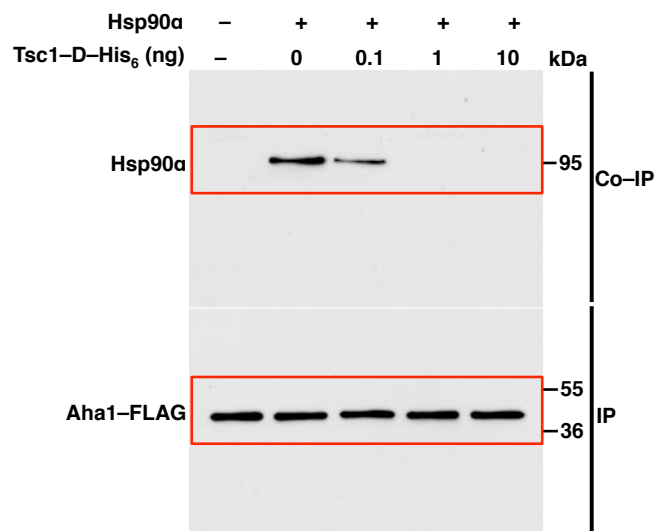

Supplement: Supplementary file 9 — Source Data for Figure 5 [file EMBJ-36-3650-s007.pdf]

# Source Data Fig 6

**A**

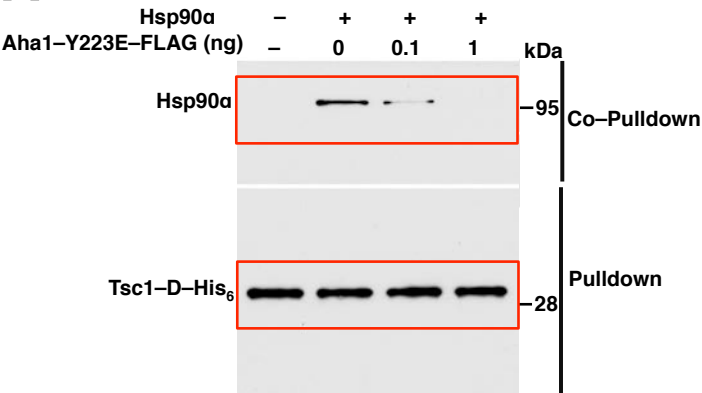

**B**

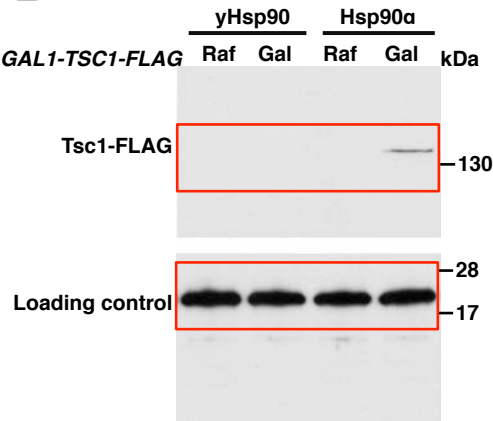

**D**

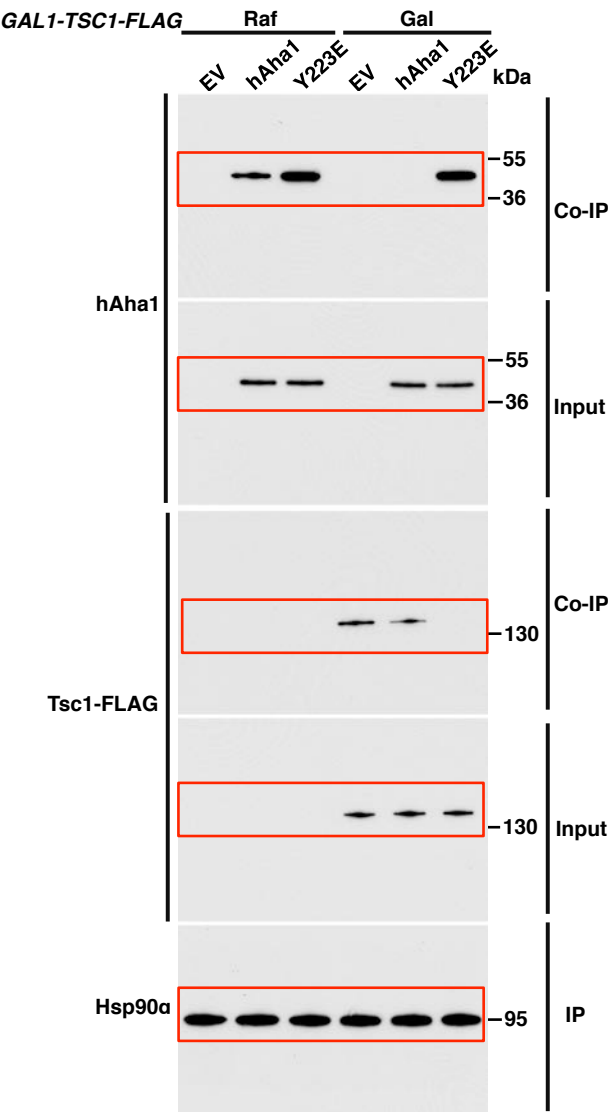

**F**

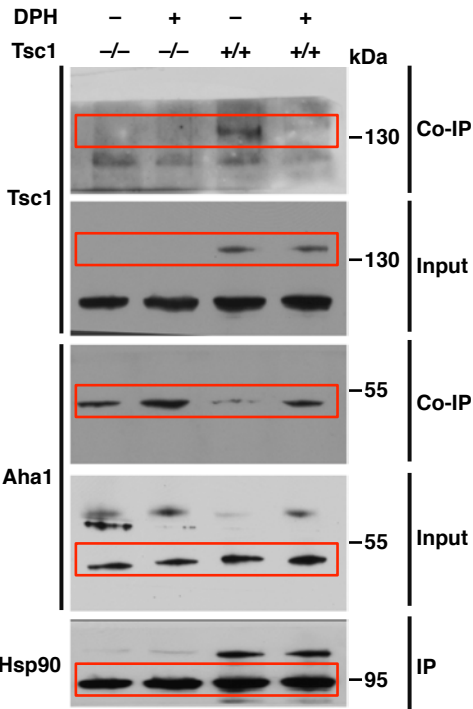

Source Data Fig 6

G

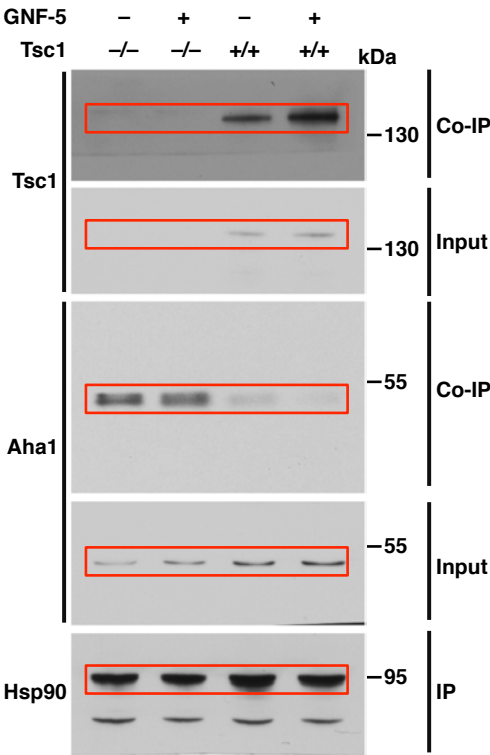

H

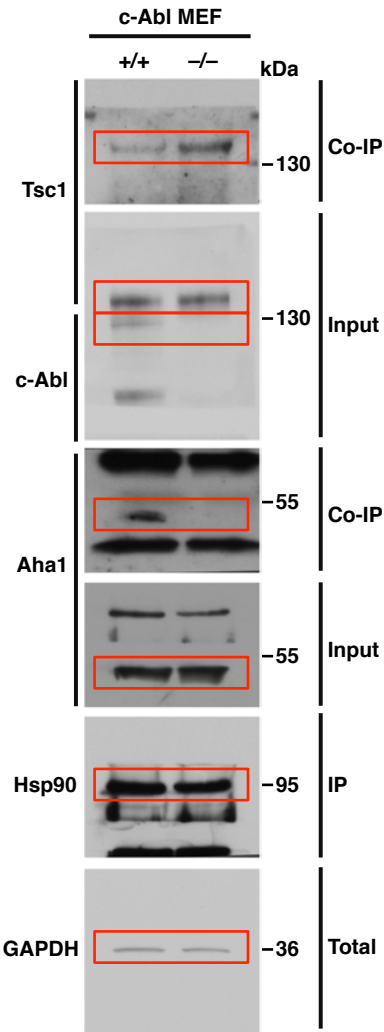

I

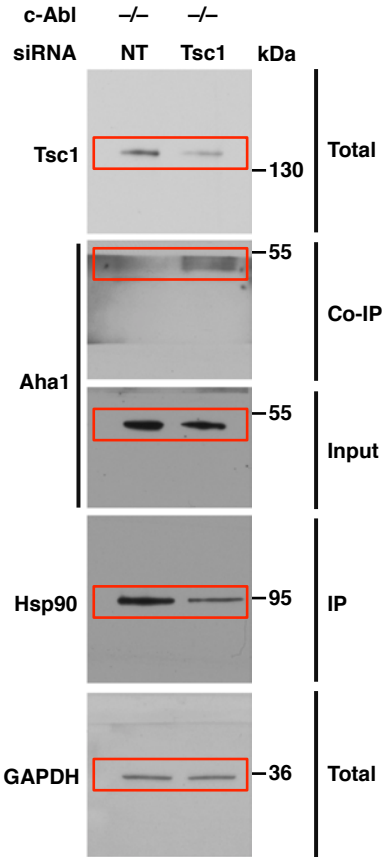

Supplement: Supplementary file 10 — Source Data for Figure 6 [file EMBJ-36-3650-s008.pdf]
